# Effect of thrombolysis with alteplase within 6 h of acute ischaemic stroke on long-term outcomes (the third International Stroke Trial [IST-3]): 18-month follow-up of a randomised controlled trial

**DOI:** 10.1016/S1474-4422(13)70130-3

**Published:** 2013-08

**Authors:** The IST-3 collaborative group

## Abstract

**Background:**

Few data are available from randomised trials about the effect of thrombolysis with alteplase on long-term functional outcome in patients who have had acute ischaemic stroke and no trial has reported effects on health-related quality of life. A secondary objective of the third International Stroke Trial (IST-3) was to assess the effect of thrombolysis on such outcomes at 18 months.

**Methods:**

In this open-label, international, multicentre, randomised, controlled trial, 3035 patients with ischaemic stroke from 12 countries were randomly allocated within 6 h of onset via a secure central system to either intravenous alteplase (0·9 mg/kg; n=1515) plus standard care or standard care alone (control; n=1520). 2348 patients were scheduled for 18-month follow-up. For our main analysis, survivors were assessed at 18 months with the Oxford handicap scale (OHS; the primary outcome was the adjusted odds of OHS score 0–2). We also used the EuroQoL (EQ) instrument and asked questions about overall functioning and living circumstances. We analysed the OHS and the five EQ domains by ordinal logistic regression and calculated the mean difference between treatment groups in EQ utility index and visual analogue scale score. Analyses were adjusted for key baseline prognostic factors. This study is registered with controlled-trials.com, number ISRCTN25765518.

**Findings:**

At 18 months, 408 (34·9%) of 1169 patients in the alteplase group versus 414 (35·1%) of 1179 in the control group had died (p=0·85). 391 (35·0%) of 1117 patients versus 352 (31·4%) of 1122 had an OHS score of 0–2 (adjusted odds ratio [OR] 1·28, 95% CI 1·03–1·57; p=0·024). Treatment was associated with a favourable shift in the distribution of OHS grades (adjusted common OR 1·30, 95% CI 1·10–1·55; p=0·002). Alteplase treatment was associated with significantly higher overall self-reported health (adjusted mean difference in EQ utility index 0·060; p=0·019). The differences between the groups in visual analogue scale score and the proportion living at home were not significant.

**Interpretation:**

IST-3 provides evidence that thrombolysis with intravenous alteplase for acute ischaemic stroke does not affect survival, but does lead to statistically significant, clinically relevant improvements in functional outcome and health-related quality of life that are sustained for at least 18 months.

**Funding:**

UK Medical Research Council, Health Foundation UK, Stroke Association UK, Research Council of Norway, AFA Insurances Sweden, Swedish Heart Lung Fund, The Foundation of Marianne and Marcus Wallenberg, Polish Ministry of Science and Education, the Australian Heart Foundation, Australian National Health and Medical Research Council, Swiss National Research Foundation, Swiss Heart Foundation, Assessorato alla Sanita (Regione dell'Umbria, Italy), and Danube University.

## Introduction

Intravenous alteplase has been approved for treatment of acute ischaemic stroke in Europe for patients who are younger than 80 years and can be treated within 4·5 h. Such use is associated with improved functional outcome at 3 months after stroke,^1^ but whether treatment improves survival and sustains functional recovery in the long term is unclear. Of the 12 completed randomised controlled trials, ten reported outcomes at 90 days or less,^1^ two reported outcomes at 6 months,^2^, ^3^ and one reported outcomes at 12 months,^3^ but none have reported effects at more than 1 year after stroke. Furthermore, the effect of thrombolysis on health-related quality of life—an important measure of the clinical and economic value of treatment—has not been reported to our knowledge.

The third International Stroke Trial (IST-3)^2^ recruited 3035 patients—half of whom were older than 80 years—to assess the effect of thrombolytic treatment with intravenous alteplase within 6 h of onset of acute ischaemic stroke. The results showed that although thrombolytic treatment was not associated with a significant difference in the proportion of patients who were alive and independent at 6 months, treatment did seem to improve functional outcome. A prespecified secondary ordinal analysis of Oxford handicap scale scores showed that treatment was associated with a favourable shift in the distribution of Oxford handicap scale scores (odds ratio [OR] 1·27, 95% CI 1·10–1·47; p=0·001).^2^ A secondary aim of IST-3 was to assess whether thrombolytic treatment improved outcomes more than 1 year after stroke, and sought to assess survival, functional outcome, health-related quality of life, overall functioning, and living circumstances at 18 months.^4^, ^5^

## Methods

### Study design and participants

The methods of the trial have been described in full previously.^2^, ^4^, ^5^, ^6^ IST-3 was a randomised, open-label trial of intravenous alteplase (0·9 mg/kg) plus standard care compared with standard care alone (control). Eligibility criteria were: symptoms and signs of clinically definite acute stroke, known time of stroke onset, treatment could be started within 6 h of onset, and exclusion by CT or MRI of intracranial haemorrhage and structural brain lesions that could mimic stroke (eg, cerebral tumour). A patient could only be included in the trial if both they (or a proxy) and their clinician believed that the treatment was promising but unproven—ie, there was neither a clear indication for treatment, nor a clear contraindication against treatment. The effect that using this uncertainty principle approach as a key eligibility criterion had on the type of patients included and excluded from the trial has been described in detail elsewhere.^2^, ^6^ Generally, patients who could be treated within licence were rarely enrolled, unless there was a specific reason that led the clinician or patient to be uncertain about whether to treat or not; as a result, 95% of enrolled patients did not meet the terms of the prevailing EU approval for treatment. All participants or proxies gave informed consent. The protocol was approved by the Multi-Centre Research Ethics Committee (Scotland) and by local ethics committees.

For the analysis presented here, we planned to assess outcome in patients who had follow-up at 6 months and 18 months. In seven countries (Austria, Belgium, Canada, Italy, Mexico, Poland, and UK) follow-up had to cease on Jan 30, 2012; therefore, we excluded any patients from these countries who were recruited after June 30, 2010, because they would not reach the 18-month follow-up point. In three countries (Australia, Norway, and Sweden), all recruited patients were to be followed up to 18 months, as part of a sub-study. Two countries (Portugal and Switzerland) followed up patients to 6 months only and were not included in this analysis.

### Randomisation

After enrolment, patients were randomly assigned by a secure central telephone or web-based computer system, which recorded baseline data and generated the treatment allocation only after the baseline data had been checked for range and consistency. The system used a minimisation algorithm to balance for key prognostic factors: geographic region, age, National Institutes of Health stroke scale score, sex, time since onset of stroke, stroke clinical syndrome, and presence or absence of visible ischaemic change on the pre-enrolment brain scan.^4^, ^5^ To avoid predictable alternation of treatment allocation, and thus potential loss of allocation concealment, patients were allocated with a probability of 0·80 to the treatment group that would minimise the difference between the groups for the key prognostic factors. Recruitment in the small double-blind phase (n=276) began in May, 2000, continued without interruption into the open-treatment phase (n=2759), and was completed in July, 2011.

### Procedures

In the ten countries participating in follow-up at 6 months and 18 months after enrolment (Australia, Austria, Belgium, Canada, Italy, Mexico, Norway, Poland, Sweden, and UK), if the patient was not known to have died, staff at each national coordinating centre contacted the patient's doctor (or hospital coordinator) to confirm that the patient was alive and that they might be approached for follow-up. In Austria and Italy, experienced stroke physicians, masked to treatment allocation, contacted all patients by telephone. In the other eight countries, IST-3 trial office staff posted a questionnaire to patients to assess outcome. Non-responders were sent a second questionnaire. If no questionnaire was returned, an experienced, masked clinician or stroke nurse assessed the patient by telephone interview. Telephone assessment of disability in stroke survivors is as valid as face-to-face interviews^7^ and postal questionnaires.^8^

The primary outcome of the trial was the proportion of patients alive and independent with an Oxford handicap scale^9^ score of 0–2 at 6 months (this outcome was chosen instead of survival alone because many people regard survival after a stroke in a disabled or dependent state as worse than death). The secondary endpoints at 18 months were: survival, Oxford handicap scale score, health-related quality of life, overall functioning, and living circumstances. The Oxford handicap scale is a six-point scale almost identical to the modified Rankin scale.^10^ In emergency care of acute ischaemic stroke, recording quality of life at baseline before randomisation was not possible; instead, quality of life was measured at 6 months and 18 months with the EuroQoL instrument,^11^ which assesses current self-rated health by a combination of questions about wellbeing and a visual analogue scale score. The questions are about the five dimensions of mobility, self-care, activity, pain or discomfort, and anxiety (the EQ-5D). Each dimension has three levels (no problems, some problems, severe problems), which can be presented individually. A unique health state is defined by combining one level from each of the five dimensions. Patients' responses can then be combined into an EQ utility index with scores ranging from −1 to +1 (where +1 represents perfect health, 0 represents a state equivalent to death, and −1 represents a state worse than death). Calculation of the EQ utility index requires valuations for all health states, and these have been estimated for the UK and other European populations.^12^ For the visual analogue scale, 100 represents the best imaginable health and 0 the worst imaginable health. We used the EuroQoL instrument because it is short and simple, and in patients with stroke it has been validated,^13^, ^14^, ^15^, ^16^, ^17^ is responsive to change,^18^ and is associated with higher response rates and fewer missing data than more complex instruments.^16^ Many patients who have had severe strokes might not be able to complete the questionnaire themselves and because responses from a proxy have reasonable validity,^15^, ^19^ we therefore accepted responses submitted by a spouse, partner, close relative, or carer.

We also assessed binary (yes or no) answers to two questions, about global functioning: “Has the stroke left you with any problems?” and activities of daily living: “Do you need help from anybody with everyday activities (in washing, dressing, feeding, and going to the toilet)?” These questions have been validated^17^ and were used previously in a large trial.^20^ We also asked whether patients were living in their own home, a relative's home, a residential home, a nursing home, or were still in hospital. Finally, the questionnaire asked patients enrolled in the open-label treatment phase what treatments they recalled being given in hospital, including thrombolysis with alteplase. If the patient or proxy did not complete a specific item on a postal questionnaire, we did not re-contact them.

### Statistical analysis

All randomly assigned patients who were due to be followed up at 18 months were included in the analysis of survival. We constructed Kaplan-Meier survival curves, and compared treatment groups with the log-rank test. Survival times were censored at 548 days after enrolment if patients died at a later date or returned an 18-month form at a later date. For patients from the Australia, Norway, Sweden, and UK, where reporting of deaths was prompt, if there was no known death date and no return of an 18-month form, patients were censored at 548 days. For patients from other countries who had no reported death date and no 18-month form, survival was censored at the date of return of the 6-month form or at the last date of contact, whichever was later. The justification for, and the methods for statistical adjustment of, the outcomes and the ordinal analyses of the Oxford handicap scale score at 18 months were specified in the statistical analysis plan and also described in the report of the primary outcomes.^2^, ^5^ We divided the Oxford handicap scale into five levels: 0, 1, 2, and 3 were retained and 4, 5, and 6 were combined into a single level. The treatment OR between one level and the next was assumed to be constant, so a single parameter (a common OR) summarises the shift in outcome distribution between treatment and control groups.

In the main analysis, we report results without imputing missing data. In the sensitivity analysis, for patients with an unknown Oxford handicap scale score at 18 months, we imputed the value from their 6-month assessment (last observation carried forward). For the EuroQoL instrument, we analysed the three levels of each EQ-5D domain as ordered categories by ordinal logistic regression, calculated the mean overall difference in visual analogue scale score between treatment groups, and estimated the EQ-5D index—calculated with a set of valuations derived from a sample of the UK population with the time trade-off method and also the UK visual analogue scale and European visual analogue scale valuations.^12^ Analyses were adjusted for baseline prognostic factors (age, National Institutes of Health stroke scale score, delay between onset and enrolment, and presence of acute ischaemic change on the baseline scan). We did several sensitivity analyses to assess the effect of missing data for Oxford handicap scale score and EQ-5D, and we assessed the effect of setting utility to zero for patients who had died. We did subgroup analyses of the effect of treatment on Oxford handicap scale score (ordinal logistic regression, as in the study by Frank and colleagues^21^) and utility subdivided by age (>80 *vs* ≤80 years), time to randomisation (≤3·0, >3·0–4·5, >4·5–6·0 h), baseline National Institutes of Health stroke scale score (0–5, 6–15, 16–25, >25), phase of the trial (masked *vs* open label), and by the person completing the form (patient *vs* proxy). For National Institutes of Health stroke scale score, we also fitted a model with baseline severity as a linear regressor with treatment-specific slopes. Analyses were done with SAS (version 9.3).

This study is registered with controlled-trials.com, number ISRCTN25765518.

### Role of the funding source

The sponsors had no role in data collection, data storage, data analysis, preparation of this report, or the decision to publish. The corresponding author had full access to all the data in the study and had final responsibility for the decision to submit for publication.

## Results

**Figure 1:**
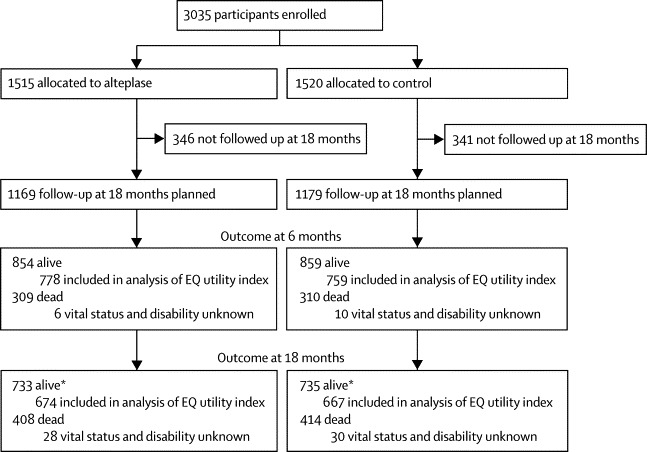
Trial profile EQ=EuroQoL. *Of the patients who were known to be alive at 18 months, 24 in the alteplase group versus 27 in the control group had a known date of death more than 18 months after enrolment, but their disability status at 18 months was unknown.

Of the 3035 patients enrolled by 156 hospitals in 12 countries, 2348 (77·4%) met the criteria for inclusion in the 18-month follow-up study—1169 assigned to alteplase, 1179 assigned to control (figure 1). The baseline characteristics of this subset were well balanced between groups (table 1) and were not much different from those who were ineligible for the 18-month follow-up analysis (appendix).

**Table 1:** Baseline characteristics of patients included in 18-month follow-up

		**Alteplase group (n=1169)**	**Control group (n=1179)**
Region			
	Americas (Canada, Mexico)	5 (<1%)	6 (1%)
	Australia	89 (8%)	90 (8%)
	Eastern Europe (Poland)	158 (14%)	159 (13%)
	Northwest Europe (UK, Austria, Belgium)	550 (47%)	556 (47%)
	Scandinavia (Norway, Sweden)	251 (21%)	250 (21%)
	Southern Europe (Italy)	116 (10%)	118 (10%)
Age			
	18–50 years	49 (4%)	57 (5%)
	51–60 years	83 (7%)	81 (7%)
	61–70 years	153 (13%)	158 (13%)
	71–80 years	291 (25%)	304 (26%)
	81–90 years	523 (45%)	512 (43%)
	>90 years	70 (6%)	67 (6%)
Women		592 (51%)	596 (51%)
National Institutes of Health stroke scale score			
	0–5	235 (20%)	236 (20%)
	6–10	323 (28%)	330 (28%)
	11–15	244 (21%)	235 (20%)
	16–20	207 (18%)	219 (19%)
	>20	160 (14%)	159 (13%)
Delay in enrolment			
	≤3·0 h	320 (27%)	307 (26%)
	>3·0–4·5 h	471 (40%)	481 (41%)
	>4·5–6·0 h	378 (32%)	389 (33%)
	>6·0 h	0 (0%)	2 (<1%)
Atrial fibrillation		347 (30%)	331 (28%)
Systolic blood pressure			
	≤143 mm Hg	380 (33%)	380 (32%)
	144–164 mm Hg	379 (32%)	405 (34%)
	≥165 mm Hg	410 (35%)	394 (33%)
Diastolic blood pressure			
	≤74 mm Hg	342 (29%)	343 (29%)
	75–89 mm Hg	409 (35%)	448 (38%)
	≥90 mm Hg	406 (35%)	381 (32%)
Blood glucose concentration*			
	≤5 mmol/L	202 (20%)	207 (20%)
	6–7 mmol/L	501 (49%)	485 (47%)
	≥8 mmol/L	324 (32%)	347 (33%)
Treatment with antiplatelet drugs in previous 48 h		599 (51%)	610 (52%)
Assessment of acute ischaemic change			
	Scan normal	99 (8%)	102 (9%)
	Scan not normal but no sign of acute change	551 (47%)	579 (49%)
	Signs of acute change	511 (44%)	490 (42%)
Predicted probability of poor outcome at 6 months†			
	<40%	633 (54%)	640 (54%)
	≥40–<50%	130 (11%)	113 (10%)
	≥50–<75%	275 (24%)	304 (26%)
	≥75%	131 (11%)	122 (10%)
Stroke syndrome			
	TACI	491 (42%)	509 (43%)
	PACI	460 (39%)	430 (36%)
	LACI	137 (12%)	133 (11%)
	POCI	79 (7%)	104 (9%)
	Other	2 (<1%)	3 (<1%)

Data are n (%). TACI=total anterior circulation infarct. PACI=partial anterior circulation infarct. LACI=lacunar infarct. POCI=posterior circulation infarct.

* Baseline glucose concentration was not recorded for the first 282 patients recruited; thus, glucose measurements were available for 2066 of 2348 participants (88%; 1027 allocated to alteplase and 1039 allocated to control).

† Calculated from a model based on age and baseline National Institutes of Health stroke scale score.^22^

**Figure 2:**
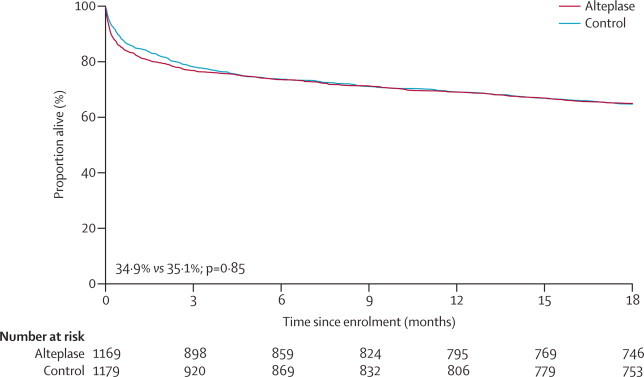
Kaplan-Meier survival curves

Of the 2348 patients scheduled for 18-month follow-up, vital status and Oxford handicap scale score at 18 months were known for 2290 (97·5%). Survival at 18 months did not differ significantly between groups: 408 of 1169 (34·9%) participants allocated to alteplase versus 414 of 1179 (35·1%) allocated to control died (log-rank p=0·85; figure 2).

At 18 months, of 2348 participants, vital status and disability were known for 2239 (95·3 %), vital status only was known for 51 (2·2%), and vital status and disability were unknown for 58 (2·5%). Oxford handicap scale scores were available for 1117 participants assigned to alteplase versus 1122 assigned to control. 391 (35·0%) patients allocated to alteplase versus 352 (31·4%) allocated to control were alive and independent (Oxford handicap scale score 0–2) at 18 months (adjusted odds ratio 1·28, 95% CI 1·03–1·57; p=0·024; unadjusted OR 1·18, 95% CI 0·99–1·40; p=0·068; table 2), with a favourable shift in Oxford handicap scale score (adjusted common OR 1·30, 95% CI 1·10–1·55; p=0·002). The size and statistical significance of the effect on Oxford handicap scale score at 18 months was robust to sensitivity analyses for missing data (data not shown). The appendix shows Oxford handicap scale score at 6 months in patients scheduled for 18-month follow-up who had data available at 6 months.

**Table 2:** Oxford handicap scale scores at 18 months

		**Alteplase group**	**Control group**	Adjusted anaylsis*		**Unadjusted analysis**†		**Difference per 1000 patients**†**(95% CI)**
				OR (95% CI)	p value	OR (95% CI)	p value	
Planned 18-month follow-up		1169	1179	..	..	..	..	..
	Missing OHS data at 18 months‡	52 (4%)	57 (5%)	..	..	..	..	..
	Number analysed (both vital and OHS status known)	1117 (96%)	1122 (95%)	..	..	..	..	..
OHS score at 18 months§								
	0	119 (11%)	83 (7%)	..	..	..	..	..
	1	135 (12%)	141 (13%)	..	..	..	..	..
	2	137 (12%)	128 (11%)	..	..	..	..	..
	3	132 (12%)	138 (12%)	..	..	..	..	..
	4	81 (7%)	107 (10%)	..	..	..	..	..
	5	105 (9%)	111 (10%)	..	..	..	..	..
Died before 18 months§¶		408 (37%)	414 (37%)	0·95 (0·78 to 1·16)	0·628	0·98 (0·83 to 1·17)	0·855	4 (−36 to 44)
Alive and independent (OHS score 0–2)§		391 (35%)	352 (31%)	1·28 (1·03 to 1·57)	0·024	1·18 (0·99 to 1·40)	0·068	−36 (−75 to 3)
Alive and had favourable outcome (OHS score 0 or 1)§		254 (23%)	224 (20%)	1·23 (0·98 to 1·55)	0·076	1·18 (0·96 to 1·44)	0·109	−28 (−62 to 6)

Data are n (%) unless stated otherwise. OHS=Oxford handicap score.

* Logistic regression of outcome on treatment group, adjusted for age, National Institutes of Health stroke scale score, and delay (all linear) and visible infarct on baseline scan.

† Standard binomial test with normal approximation.

‡ Includes patients who did not return an 18-month form but died more than 18 months after enrolment (figure 1).

§ Percentages based on number analysed for OHS. For one participant, OHS was imputed on the basis of responses to EQ-5D.

¶ If all patients known to be alive are included in the denominators, the percentage dead at 18 months are 35·8% in the alteplase group and 36·0% in the control group.

The EQ utility index could be calculated for 1341 (91·3%) of the 1468 patients who were alive at 18 months. 591 (44%) of these assessments were completed by patients themselves, 724 (54%) by a valid proxy, and 25 (2%) by a doctor. Treatment was associated with significant improvements in mobility, self-care, ability to do usual activities, and pain or discomfort, with no evidence of an effect on anxiety or depression (table 3). At 18 months, alteplase was associated with significantly fewer patients reporting being left with problems and needing help with everyday activities (table 3).

**Table 3:** EQ-5D and other assessments of function at 18 months

		**Alteplase group**	**Control group**	Odds ratio (95% CI)*	**p value**	**Difference per 1000 patients**†**(95% CI)**
**EQ-5D**						
Mobility		702	692	..	..	..
	No problems walking	283 (40%)	259 (37%)	1·30 (1·05 to 1·61)	0·017	−29 (−80 to 22)
	Some problems walking	343 (49%)	346 (50%)	..	..	11 (−41 to 64)
	Confined to bed	76 (11%)	87 (13%)	..	..	17 (−16 to 51)
Self-care		695	689	..	..	..
	No problems with self-care	372 (54%)	328 (48%)	1·43 (1·16 to 1·78)	0·001	−59 (−112 to −7)
	Some problems washing or dressing	176 (25%)	191 (28%)	..	..	24 (−23 to 70)
	Unable to wash or dress	147 (21%)	170 (25%)	..	..	35 (−9 to 79)
Usual activities		699	694	..	..	..
	No problems with usual activities	235 (34%)	209 (30%)	1·32 (1·07 to 1·62)	0·008	−35 (−84 to 14)
	Some problems with usual activities	258 (37%)	256 (37%)	..	..	0 (−51 to 50)
	Unable to do usual activities	206 (29%)	229 (33%)	..	..	35 (−13 to 84)
Pain or discomfort		698	694	..	..	..
	No pain or discomfort	344 (49%)	304 (44%)	1·26 (1·02 to 1·56)	0·029	−55 (−107 to −2)
	Moderate pain or discomfort	316 (45%)	355 (51%)	..	..	59 (6 to 111)
	Extreme pain or discomfort	38 (5%)	35 (5%)	..	..	−4 (−27 to 19)
Anxiety or depression		693	690	..	..	..
	Not anxious or depressed	353 (51%)	349 (51%)	1·05 (0·85 to 1·29)	0·668	−4 (−56 to 49)
	Moderately anxious or depressed	292 (42%)	290 (42%)	..	..	−1 (−53 to 51)
	Extremely anxious or depressed	48 (7%)	51 (7%)	..	..	5 (−23 to 32)
**Additional questions about overall function**						
Stroke left patient with problems		484/700 (69%)	542/699 (78%)	1·67 (1·30 to 2·17)	<0·0001	84 (38 to 130)
Needs help with everyday activities		298/696 (43%)	350/692 (51%)	1·59 (1·25 to 2·00)	<0·0001	78 (25 to 130)

Data are n (%) unless stated otherwise.

* Logistic regression of outcome on treatment group, adjusted for age, National Institutes of Health stroke scale score, and delay (all linear) and visible infarct on baseline scan.

† Standard binomial test with normal approximation.

Although treatment with alteplase was associated with a significantly higher EQ utility index in survivors (p=0·028; table 4), the mean adjusted difference in visual analogue scale score was not significant (p=0·072; table 4). These findings were robust in the sensitivity analyses (data not shown). The appendix shows EQ-5D, EQ utility index, and visual analogue scale score at 6 months and 18 months using different valuations. Of the participants who were still alive, the proportion who were resident at home did not differ significantly between groups (appendix).

**Table 4:** EQ utility index and visual analogue scale score assessment of overall health at 18 months

	**Alteplase group**		**Control group**		Adjusted analysis*		**Unadjusted analysis**†	
	n	Mean (SE)	n	Mean (SE)	Mean difference (SE)	p value	Mean difference (SE)	p value
Visual analogue scale score	653	62·07 (0·90)	648	60·57 (0·91)	2·18 (1·21)	0·072	1·49 (1·28)	0·244
EQ utility index	674	0·550 (0·015)	667	0·502 (0·016)	0·062 (0·020)	0·002	0·049 (0·022)	0·028

* Adjusted for age, National Institutes of Health Stroke Scale score, delay from onset to enrolment, and presence of visible ischaemia on the baseline scan.

† Significance based on *t* test. Utility based on UK time trade-off valuations on a scale of −1 to +1.

For the ordinal subgroup analysis of Oxford handicap scale score at 18 months, significant interactions existed between baseline variables and treatment effect. Greater differences in favour of alteplase were reported for age older than 80 years (p=0·032) and high National Institutes of Health stroke scale score (p=0·021), but not for time to treatment, respondent (patient *vs* proxy), or masking of assessment of outcome (double blind *vs* open label; appendix). When age, delay, and National Institutes of Health stroke scale score were treated as continuous variables, the interaction of ordinal Oxford handicap scale score with age became non-significant, delay remained non-significant, and for National Institutes of Health stroke scale score the p value for a trend was 0·004 (appendix). For EQ utility index, when subgroups were in discrete categories, none of the interactions were statistically significant (appendix). However, when the National Institutes of Health stroke scale score was treated as continuous, every five-point increase in score reduced the EQ utility index by 0·12 in the alteplase group versus 0·15 in the control group (adjusted estimates; p=0·008 for difference in slopes). For delay in enrolment time and age there was no trend in EQ utility index, irrespective of whether the variables were grouped or entered into models as a linear trend (data not shown).

Of the 1468 patients who were alive at 18 months, 1260 were asked to recall if they had been given thrombolytic treatment (appendix); 273 in the alteplase group versus 156 in the control group correctly recalled whether or not they had received thrombolytic treatment. In both treatment groups, the ability to recall treatment correctly was associated with better outcome; patients with correct recall were more likely to have an Oxford handicap scale score of 0–2 than were those who remembered incorrectly or did not know (62·5% *vs* 49·3%; 0·0001). Of patients with correct recall, those treated with alteplase were more likely to have an Oxford handicap scale score of 0–2 than were those in the control group (66·7% *vs* 55·1%; 0·018), whereas of those who did not remember correctly, outcomes did not differ significantly between groups (OHS 0–2 48·6% *vs* 49·9%; 0·714); a significant interaction existed between recall status and treatment (p<0·0001).

## Discussion

We have shown that, for treatment of acute ischaemic stroke, thrombolysis with intravenous alteplase seems to provide a benefit at 18 months. Treatment had no effect on survival, but was associated with a significant increase in the likelihood of being alive and independent. However, the unadjusted absolute difference in the number of patients alive and independent at 18 months was not significant, so judgment on whether or not the results are clinically significant rests on the quality of the data and the overall patterns of effect seen across all measures. The ordinal estimates of effect at 6 months and 18 months were similar and significant. Treatment was also associated with a gain in health-related quality of life that was significant for four of the five dimensions of the EQ-5D and the overall EQ utility index (though not for visual analogue scale score). Living circumstances did not differ significantly between groups.

Strengths of this study are the large number of patients and the completeness of follow-up. Of the patients scheduled for 18-month follow-up, a small proportion were missing data for both vital and functional outcome status. We estimated the EQ utility index in more than 91% of survivors (a similar proportion to that in a trial^23^ of younger and less impaired patients with coronary artery disease) and our sensitivity analyses also showed that the estimates of overall health-related quality of life with the EQ utility index were robust to various assumptions about missing data. Although thrombolytic treatment was associated in survivors with less functional impairment, better health-related quality of life, and less likelihood of being left with problems and needing help with daily activities after stroke, it did not translate into a higher proportion of patients living at home at 18 months, perhaps because living circumstances are affected by social and financial factors that are not influenced by treatment. We believe that the direction and size of the effects are clinically significant and will inform health economic assessments of thrombolytic treatment. For example, in 2002, the estimated cost of long-term care of an independent survivor of stroke was £876 per year and that of a dependent survivor was £11 292 per year,^24^ so even a small difference in the proportion of patients who survive and are independent will have substantial economic impact.

Lyden^25^ has identified limitations of IST-3, chiefly that treatment was not masked. Patient-reported outcomes—eg, health-related quality of life—are subjective,^26^ and recall of thrombolytic treatment could affect patient responses. Only 30% of survivors correctly recalled whether or not they had received thrombolytic treatment. As expected, accurate recall was associated with better outcome in both treatment groups. Thus, recall bias might have affected our findings. However, the analysis of recall was based on a variable measured in a subset of survivors after randomisation and so could itself be biased. The effects of treatment on the Oxford handicap scale score and EQ utility index were much the same in the masked and open-label parts of the study (appendix). Assessment of health-related quality of life is limited because many patients who have had a stroke are unable to complete the form themselves. The high proportion of forms completed by a proxy in IST-3 is a result of the severity of stroke in the patients included in the trial. Although the use of surrogates is a potential weakness, it did enable us to achieve satisfactory response rates; however, because proxies tend to assign worse health status than do patients,^15^ we were reassured that there was no interaction between the person responding and the effect of treatment on utility or Oxford handicap scale score. Not all enrolled patients were scheduled to be followed up for 18 months, but the selection criteria for the longer follow-up cohort did not seem to introduce relevant imbalances at baseline, nor were the characteristics of the cohort substantially different from those not included in long-term follow-up. We therefore believe the 18-month follow-up cohort is representative of the trial as a whole.

Another weakness is that the trial was under-powered, so the subgroup analyses of the effects of baseline age, stroke severity, and delay to enrolment on the Oxford handicap scale score and health-related quality of life should be treated with caution. These are secondary analyses of a secondary outcome, and the apparent lack of effect of time to treatment might be due to chance. Furthermore, a more appropriate assessment of the complex interactions between age, stroke severity, and time to treatment will be available from a meta-analysis of individual patient data by the Stroke Thrombolysis Trialists.^27^

In conclusion, IST-3 adds to the evidence from previous trials (panel) and shows that although thrombolysis for acute ischaemic stroke with intravenous alteplase does not improve survival, there is evidence of improvement in several measures of function and quality of life in survivors of all ages for up to 18 months after treatment.

Panel **Research in context**

**Systematic review**

The primary results of IST-3^2^ included a systematic review of randomised controlled trials of alteplase in acute stroke.^1^ To accompany this review we searched up to April 30, 2013, for additional randomised trials of intravenous alteplase versus control within 6 h of onset of acute stroke in the Cochrane Stroke Trials Registry, Internet Stroke Trials Centre, and reference lists in review articles and conference abstracts. For the Cochrane Stroke Trials Registry we searched for interventions with thrombolytic drugs in acute ischaemic stroke added since the last update of the Cochrane review. For the Internet Stroke Center, we searched for “acute ischemic stroke”, “acute ischaemic stroke”, “thrombolysis”, “thrombolytic therapy”, “alteplase”, and “recombinant tissue plasminogen activator”. For each trial, we checked the primary trial publication, and when available, the trial protocol, to determine if it was planned to collect long-term clinical outcome data (ie, more than 90 days after enrolment) or health-related quality-of-life data, as assessed by a valid instrument such as EQ-5D or Short Form 36.

Of the 12 completed randomised controlled trials, ten reported outcome at 90 days or less,^1^ two reported clinical outcome at 6 months^2^, ^3^ and one at 12 months,^3^ but none reported effects more than 12 months after stroke. The Second European Collaborative Acute Stroke Study collected data on health-related quality of life at 90 days with the SF-36, but has yet to report those data. In the NINDS Trial,^3^ mortality at 12 months did not differ significantly between alteplase and placebo groups (24% *vs* 28%; p=0·29). The primary outcome was favourable outcome, defined as minimal or no disability as measured by the Barthel index, the modified Rankin scale, and the Glasgow outcome scale, and the treatment effect was assessed with a global statistic. The global statistic favoured the alteplase group at 6 months (OR for a favourable outcome 1·7, 95% CI 1·3–2·3) and at 12 months (1·7, 1·2–2·3).

**Interpretation**

IST-3 confirms the evidence from previous trials on the neutral effect of thrombolysis with alteplase on survival after stroke in a much larger sample, and adds to the evidence that improvements in function reported at earlier timepoints are evident at 18 months. IST-3 also provides the first validated estimates of the effect of thrombolysis with alteplase on health-related quality of life.

Correspondence to: Prof Peter Sandercock, Division of Clinical Neurosciences, University of Edinburgh, Western General Hospital, Crewe Road, Edinburgh EH4 2XU, UK peter.sandercock@ed.ac.uk
