# Supplementary material for: Effect of thrombolysis with alteplase within 6 h of acute ischaemic stroke on long-term outcomes (the third International Stroke Trial [IST-3]): 18-month follow-up of a randomised controlled trial
Source: Lancet Neurol. 2013 Aug;12(8):768–76. doi: 10.1016/S1474-4422(13)70130-3 (PMC3854835; doi:10.1016/S1474-4422(13)70130-3)
Supplement: Supplementary appendix [file mmc1.pdf]

THE LANCET Neurology

Supplementary webappendix

This webappendix formed part of the original submission and has been peer reviewed. We post it as supplied by the authors.

Supplement to: The IST-3 collaborative group. Effect of thrombolysis with alteplase within 6 h of acute ischaemic stroke on long-term outcomes (the third International Stroke Trial [IST-3]): 18-month follow-up of a randomised controlled trial. *Lancet Neurol* 2013; published online June 21. http://dx.doi.org/10.1016/S1474-4422(13)70130-3.

Supplementary appendix to
Effect of thrombolysis with recombinant tissue plasminogen activator for acute ischaemic stroke on long-term outcomes in the third international stroke trial (IST-3) : a randomised controlled trial

The IST-3 Collaborative Group

Contents of appendix

Supplementary Tables

1. Comparison of baseline characteristics of patients scheduled for 18 month follow –up versus those not scheduled for such follow-up
2. OHS at six months for patients with planned 18 month follow-up and known disability status
3. EQ-5D and additional questions at six months (in planned 18 month follow-up population)
4. Treatment group difference in EQ utility and VAS index at six and 18 months: effects of different valuations
5. Living circumstances at six and eighteen months
6. Effects of treatment in key subgroups on ordinal analysis of OHS at 18 months
7. Effects of treatment in key subgroups on EQ utility index at 18 months
8. Recall at six and 18 months of thrombolytic drug administration on day of admission

Committees and list of members of the IST-3 collaborative group.

Web Table 1 Comparison of baseline characteristics of patients scheduled for 18-month follow –up versus those not scheduled for such follow-up

	Planned 18-month follow-up		No planned 18-month follow-up	
	**No.**	**(%)**	**No.**	**(%)**
**Age in years**				
18-50	106	(5%)	21	(3%)
51-60	164	(7%)	38	(6%)
61-70	311	(13%)	54	(8%)
71-80	595	(25%)	129	(19%)
81-90	1035	(44%)	372	(54%)
over 90	137	(6%)	73	(11%)
**Female**	1188	(51%)	382	(56%)
**NIHSS**				
0 to 5	471	(20%)	141	(21%)
6 to 10	653	(28%)	199	(29%)
11 to 15	479	(20%)	122	(18%)
16 to 20	426	(18%)	117	(17%)
> 20	319	(14%)	108	(16%)
**Delay in randomisation**				
0-3 hours	627	(27%)	222	(32%)
3-4.5 hours	952	(41%)	225	(33%)
4.5-6 hours	767	(33%)	240	(35%)
>6 hours	2	(0%)	0	(.%)
**Atrial Fibrillation**	678	(29%)	236	(34%)
**Systolic BP**				
<= 143 mmHg	760	(32%)	219	(32%)
144 - 164 mmHg	784	(33%)	232	(34%)
>= 165 mmHg	804	(34%)	236	(34%)
**Diastolic BP**				
<= 74 mmHg	685	(29%)	222	(32%)
75 - 89 mmHg	857	(37%)	272	(40%)
>= 90 mmHg	787	(34%)	193	(28%)
**Blood glucose**[1]				
<= 5 mmol/l	409	(20%)	130	(19%)
6 - 7 mmol/l	986	(48%)	316	(46%)
>= 8 mmol/l	671	(32%)	240	(35%)
**Treatment with antiplatelet drugs in previous 48hrs**	1209	(51%)	353	(51%)
**Expert reader's assessment of acute ischaemic change**				
Scan completely normal	201	(9%)	69	(10%)
Scan not normal but no sign of acute change	1130	(48%)	400	(58%)
Signs of acute change	1001	(43%)	216	(32%)
**Predicted probability of poor outcome at 6 months**[2]				
< 40%	1273	(54%)	378	(55%)
40% - 50%	243	(10%)	83	(12%)
50% - 75%%	579	(25%)	163	(24%)
>= 75%	253	(11%)	63	(9%)
**Stroke syndrome**				
TACI	1000	(43%)	306	(45%)
PACI	890	(38%)	256	(37%)
LACI	270	(11%)	62	(9%)
POCI	183	(8%)	63	(9%)
OTHER	5	(0%)	0	(.%)

NIHSS = National Institutes of Health Stroke Scale, TACI= Total Anterior Circulation Infarct, PACI = Partial Anterior Circulation Infarct, LACI = Lacunar Infarct, POCI = Posterior Circulation Infarct

1. For the first 282 patients recruited in the study, pre-randomisation glucose was not recorded; as a result, glucose measurements were available for 2066/2348 (88%).
2. Probability of a poor outcome calculated from a model based on age and baseline NIHSS[27]

Web Table 2 OHS at six months for patients with planned 18-month follow-up and known disability status

	rt-PA		Control		Adjusted analysis[1]		Unadjusted analysis[2]		(Control - rt-PA) difference per 1000[3]
	No.	(%)	No.	(%)	OR rt-PA:Control (95% CI)	P	OR rt-PA:Control (95% CI)	P	(95% CI)
No. planned 18 month follow-up	1169		1179						
Missing 6 month status[4]	29		41						
No. for 6 month analysis[5]	1140		1138						
0	115	(10%)	89	(8%)					
1	170	(15%)	155	(14%)					
2	157	(14%)	173	(15%)					
3	184	(16%)	154	(14%)					
4	82	(7%)	105	(9%)					
5	152	(13%)	193	(17%)					
Died before 6 months[6]	309	(27%)	310	(27%)	0.94 (0.76 , 1.16)	0.554	0.99 (0.83 , 1.19)	0.942	1 (-35, 38)
Alive and independent (0+1+2)	437	(38%)	409	(36%)	1.18 (0.97 , 1.45)	0.101	1.11 (0.93 , 1.31)	0.237	-24 (-64, 16)
Alive and favourable outcome (0+1)	284	(25%)	244	(21%)	1.29 (1.04 , 1.61)	0.022	1.22 (1.00 , 1.48)	0.050	-35 (-69, -0)

1 Logistic regression of outcome on treatment group, adjusted for age, NIHSS and delay (all linear) and visible infarct on baseline scan. For OHS an ordinal analysis was also performed (with levels 0, 1, 2, 3 discrete and 4+5+6 grouped): adjusted common OR = 1·35 (95%CI 1·15 -1·59, p=0·0004)

2 Logistic regression of outcome on treatment group

3 Standard binomial test with normal approximation

4 Patients who did not return a 6 month form· Vital status at 6 months was known for all members of 18 month follow-up cohort·

5 The percentages by OHS category are based on these totals·

6 If all patients known to be alive (see note 4) are included in denominators percents dying by 6 months are : rt-PA 26·4%, Control 26·3%

Web Table 3 : EQ-5D and additional questions at six months (in planned 18-month follow-up population)

	rt-PA<br>No (%)	Control<br>No· (%)	Odds Ratio (95% CI)[1]	p	(Control - rt-PA) difference per 1000 (95% CI) [2]
Mobility (N= 815 rt-PA, 814 Control)					
No problems walking	320 (39%)	313 (38%)	1·14 (0·94 - 1·38)	0·189	-8 (-55, 39)
Some problems walking	393 (48%)	386 (47%)			-8 (-57, 41)
Confined to bed	102 (13%)	115 (14%)			16 (-17, 49)
Self-care (N= 819 rt-PA, 813 Control)					
No problems with self care	429 (52%)	394 (48%)	1·37 (1·13 - 1·67)	0·002	-39 (-88, 9)
Some problems washing or dressing	233 (28%)	211 (26%)			-25 (-68, 18)
Unable to wash or dress self	157 (19%)	208 (26%)			64 (24, 104)
Usual activities (N= 816 rt-PA, 804 Control)					
No problems with usual activities	271 (33%)	231 (29%)	1·39 (1·15 - 1·68)	0·001	-45 (-90, 0)
Some problems with usual activities	299 (37%)	288 (36%)			-8 (-55, 39)
Unable to perform usual activities	246 (30%)	285 (35%)			53 (7, 99)
Pain or discomfort (N= 806 rt-PA, 802 Control)					
No pain or discomfort	389 (48%)	381 (48%)	1·03 (0·85 - 1·25)	0·74	-8 (-56, 41)
Moderate pain or discomfort	366 (45%)	374 (47%)			12 (-36, 61)
Extreme pain or discomfort	51 (6%)	47 (6%)			-5 (-28, 19)
Anxiety or depression (N= 808 rt-PA, 801 Control)					
Not anxious or depressed	395 (49%)	377 (47%)	1·10 (0·91 - 1·33)	0·330	-18 (-67, 31)
Moderately anxious or depressed	344 (43%)	350 (44%)			11 (-37, 60)
Extremely anxious or depressed	69 (9%)	74 (9%)			7 (-21, 35)
**Additional questions on overall function**					
Stroke left patient with problems (N= 827 rt-PA, 821 Control)	596 (72%)	648 (79%)	1·54[3](1·20 – 1·92)	<0·0001	69 (27, 110)
Needs help with everyday activities (N= 827 rt-PA, 820 control)	387 (47%)	423 (52%)	1·32[3] (1·06 - 1·64)	0·011	48 (0, 96)

1 Logistic regression of outcome on treatment group, adjusted for age, NIHSS and delay (all linear) and visible infarct on baseline scan; odds ratio > 1 indicates treatment associated with greater odds of a lesser degree of problems

2 Standard binomial test with normal approximation

3. Odds ratio > 1 indicates increases odds of not being left with problems or needing help

Web Table 4 Treatment group difference in EQ utility and VAS index at six and 18 months: effects of different valuations

	Adjusted differences						Unadjusted differences					
	UK TTO		UK VAS		European VAS		UK TTO		UK VAS		European VAS	
	Mean (SE)	P	Mean (SE)	P	Mean (SE)	P	Mean (SE)	P	Mean (SE)	P	Mean (SE)	P
EQ-5D Utility at Six months	0·040 (0·019)	0·033	0·031 (0·014)	0·025	2·9 (1·3)	0·024	0·031 (0·021)	0·150	0·024 (0·016)	0·129	2·2 (1·4)	0·123
EQ-5D Utility at Six months (survivors to 18 months)	0·044 (0·019)	0·022	0·033 (0·015)	0·024	3·0 (1·3)	0·024	0·032 (0·022)	0·131	0·024 (0·017)	0·147	2·2 (1·5)	0·142
EQ-5D Utility at Eighteen months	0·060 (0·019)	0·002	0·047 (0·015)	0·002	4·3 (1·3)	0·001	0·049 (0·022)	0·028	0·038 (0·017)	0·025	3·5 (1·5)	0·024
Change : Eighteen minus Six months	0·007 (0·015)	0·650	0·008 (0·011)	0·478	0·7 (1·0)	0·462	0·005 (0·015)	0·735	0·007 (0·011)	0·551	0·6 (1·0)	0·535

TTO= Time trade-off, VAS= Visual analogue scale· The valuations are described and reported in EQ-5D value sets: Inventory, comparative review and user guide[12]

Web Table 5 Living circumstances at six and 18 months

	rt-PA		Control	
	**N**	**%**	**N**	**%**
**6 months**				
Own home	639	76·9	626	75·6
Relative's home	49	5·9	56	6·8
Residential home	45	5·4	41	5·0
Nursing home	93	11·2	96	11·6
Still in hospital	5	0·6	9	1·1
All	831	100·0	828	100·0
**18 months**				
Own home	574	81·0	553	78·2
Relative's home	29	4·1	40	5·7
Residential home	45	6·3	42	5·9
Nursing home	60	8·5	69	9·8
Still in hospital	1	0·1	3	0·4
All	709	100·0	707	100·0

Web Table 6. Adjusted subgroup effects on OHS at 18 months: Ordinal analysis

			Ordinal analysis			
	Alive and independent / Total		Adjusted [1]		Unadjusted	
	rt-PA	Control	OR (99% CI) [2]	P [3]	OR (99% CI) [2]	P[3]
**Age [4]**				·		·
**<= 80 years**	243/547 (44%)	249/569 (44%)	1·10 (0·82, 1·47)	0·032	1·07 (0·81, 1·42)	0·123
**> 80 years**	148/570 (26%)	103/553 (19%)	1·57 (1·11, 2·23)	·	1·38 (1·00, 1·90)	·
**Delay [4]**				·		·
**<= 3**	92/308 (30%)	63/300 (21%)	1·54 (0·96, 2·47)	0·492	1·47 (0·95, 2·26)	0·266
**>3 - =<4·5**	144/450 (32%)	145/449 (32%)	1·16 (0·81, 1·66)	·	1·06 (0·77, 1·48)	·
**>4·5 - =<6**	155/359 (43%)	144/371 (39%)	1·32 (0·91, 1·92)	·	1·15 (0·81, 1·63)	·
**NIHSS [4]**				·		·
**0 to 5**	157/224 (70%)	152/224 (68%)	1·23 (0·80, 1·90)	0·021	1·20 (0·78, 1·85)	0·025
**6 to 15**	194/539 (36%)	185/535 (35%)	1·14 (0·84, 1·54)	·	1·07 (0·80, 1·44)	·
**16 to 24**	35/293 (12%)	15/315 (5%)	2·33 (1·28, 4·25)	·	1·99 (1·13, 3·49)	·
**>= 25 [5]**	5/61 (8%)	0/48 (0%)	7·81 (0·82, 74·1)	·	5·21 (0·67, 40·8)	·
**Respondent**				·		·
**Patient**	263/318 (83%)	240/308 (78%)	1·36 (0·94, 1·97)	0·551	1·26 (0·87, 1·82)	0·631
**Proxy**	128/390 (33%)	111/401 (28%)	1·59 (1·12, 2·27)	·	1·32 (0·94, 1·85)	·
**Trial phase**				·		·
**Blinded**	34/131 (26%)	31/136 (23%)	1·29 (0·64, 2·58)	0·955	1·17 (0·61, 2·22)	0·994
**Open**	357/986 (36%)	321/986 (33%)	1·30 (1·02, 1·65)	·	1·17 (0·94, 1·46)	·

1 Analysis by age adjusted for linear effects of delay and NIHSS; analysis by delay adjusted for linear effects of age and NIHSS; analysis by NIHSS adjusted for linear effects of age and delay; analysis by proxy group and trial phase adjusted for linear effects of age, NIHSS and delay. Additionally all analyses adjusted for visible ischaemia on baseline scan.

2 Odds ratio for effect of rtPA on an improvement shift of one level in OHS where this scale has been grouped into 5 levels (see text).

3 Wald test of interaction between treatment and subgroup factor.

4 Trend tests of treatment interaction give P=0·24, 0·004 and 0·41 for age, NIHSS and delay respectively, in each case adjusting for the other variables· Unadjusted trend tests give P=0·67, 0·006 and 0·34 respectively.

5 Logistic model for this subgroup has quasi-complete separation, hence OR estimates are unreliable.

Web Table 7. Adjusted subgroup effects on EQ utility index at 18 months

	Mean Utility (SE) [1]		Adjusted Analysis [2]			Unadjusted Analysis [3]	
	rt-PA	Control	Adjusted difference (SE)	P	Interaction test [4]	Unadjusted difference (SE)	P
**Age [5]**					·		
**<= 80 years**	0·599 (0·020)	0·558 (0·020)	0·045 (0·025)	0.071	0·277	0·041 (0·028)	0·141
**> 80 years**	0·480 (0·024)	0·409 (0·026)	0·087 (0·033)	0.009	·	0·071 (0·035)	0·043
**Delay [5]**					·		
**<= 3**	0·475 (0·033)	0·423 (0·036)	0·081 (0·046)	0.080	0·717	0·051 (0·049)	0·297
**>3 - =<4·5**	0·536 (0·025)	0·502 (0·026)	0·033 (0·032)	0.293	·	0·034 (0·036)	0·344
**>4·5 - =<6**	0·617 (0·023)	0·546 (0·023)	0·081 (0·030)	0.008	·	0·071 (0·033)	0·030
**NIHSS [5]**					·		
**0 to 5**	0·726 (0·024)	0·715 (0·020)	0·017 (0·031)	0.580	0·111	0·012 (0·031)	0·707
**6 to 15**	0·537 (0·021)	0·499 (0·022)	0·055 (0·030)	0.070	·	0·039 (0·030)	0·198
**16 to 24**	0·342 (0·037)	0·200 (0·035)	0·146 (0·050)	0.004	·	0·142 (0·050)	0·005
**>= 25**	0·212 (0·089)	0·018 (0·137)	0·251 (0·164)	0.140	·	0·194 (0·168)	0·258
**Respondent**					·		
**Patient**	0·759 (0·015)	0·737 (0·016)	0·026 (0·021)	0.217	0·403	0·023 (0·022)	0·307
**Proxy**	0·378 (0·021)	0·324 (0·021)	0·072 (0·028)	0.011	·	0·054 (0·030)	0·072
**Trial phase**					·		
**Blinded**	0·469 (0·051)	0·386 (0·052)	0·087 (0·068)	0.200	0·487	0·084 (0·073)	0·253
**Open**	0·560 (0·016)	0·516 (0·016)	0·059 (0·021)	0.005	·	0·043 (0·023)	0·060

1. Utility determined from EQ5D responses using UK valuations on time trade-off basis.
2. Linear regression of utility on treatment plus adjustment factors. Analysis by age adjusted for linear effects of delay and NIHSS; analysis by delay adjusted for linear effects of age and NIHSS; analysis by NIHSS adjusted for linear effects of age and delay; analysis by proxy group and trial phase adjusted for linear effects of age, NIHSS and delay. Additionally, all analyses adjusted for visible ischaemia on baseline scan.
3. t-tests of difference in mean utility between treatment groups within each subgroup.
4. F test of interaction between treatment and subgroup factor in linear model with adjustment variables.
5. Trend tests of treatment interaction give P=0·21, 0·008 and 0·56 for age, NIHSS and delay respectively.

Web Table 8 Recall at 18 months of thrombolytic drug administration on day of hospital admission among the cohort scheduled for 18-month follow-up

	rt-PA		control		All	
Recall of thrombolytic therapy	No.	% OHS 0-2	No.	% OHS 0-2	No.	% OHS 0-2
Remembered treatment correctly	273	66.7	156	55.1	429	62.5
Remembered incorrectly or did not know	360	48.6	471	49.9	831	49.3
Question not asked or in double-blind phase[1]	76	44.7	81	38.3	157	41.4

1. The question about recall of treatment was first asked towards the end of the double blind phase of the trial. Some patients who were recruited in the open phase were not sent the correct questionnaire, and so were inadvertently not asked the question

Committees and List of members of IST-3 collaborative group

Trial Steering Committee:
Independent Chairman, Professor Colin Baigent (University of Oxford), Professor David Chadwick (Former Chairman, Retired)): University of Liverpool; Independent members: Dr Pippa Tyrrell (Manchester University), Professor Gordon Lowe (Glasgow University); Co-principal Investigators: Professor Peter Sandercock (University of Edinburgh); Professor Richard Lindley (Sydney Medical School – Westmead Hospital, and the George Institute for Global Health, University of Sydney); Chief Investigator for Neuroradiology: Professor Joanna Wardlaw (University of Edinburgh); Professor Martin Dennis (University of Edinburgh); Statistician: Geoff Cohen; Trial Co-ordinator: Karen Innes. Lay representative: Heather Goodare.

CT and MR reading panel
Joanna Wardlaw, Andrew Farrall, Rüdiger von Kummer, Lesley Cala, Anders von Heijne, Zoe Morris, Alessandro Adami, Andre Peeters, Gillian Potter, Nick Brady.

Data Monitoring Committee
Professor Rory Collins, Oxford University, UK (Chairman), Professor Philip Bath (Nottingham University), Professor Jan van Gijn (former member, now retired) (University of Utrecht, The Netherlands), Professor Richard Gray (University of Oxford), Professor Robert Hart (McMaster University, Canada), Professor Salim Yusuf (McMaster University, Canada).

Event Adjudication Committee
Keith Muir, Peter Sandercock, Richard Lindley.

National Co-ordinators and Associate National Co-ordinators
Australia: Richard Lindley, Graeme J. Hankey. Austria: Karl Matz, Michael Brainin. Belgium: Andre Peeters. Canada: Gord Gubitz, Stephen Phillips. Italy: Stefano Ricci. Mexico: Antonio Arauz. Norway: Eivind Berge, Karsten Bruins Slot. Poland: Anna Czlonkowska, Adam Kobayashi. Portugal: Manuel Correia. Switzerland: Phillippe Lyrer, Stefan Engelter. Sweden: Veronica Murray, Bo Norrving, Andreas Terént, Per Wester: UK: Graham Venables.

Trial Coordinating Centre, Edinburgh. Karen Innes, Alison Clark, David Perry, Vera Soosay, David Buchanan, Sheila Grant, Eleni Sakka, Jonathan Drever, Pauli Walker, Indee Herath, Ann Leigh Brown, Paul Chmielnik, Christopher Armit, Andrea Walton, Mischa Hautvast, Steff Lewis, Graeme Heron, Sylvia Odusanya, Pam Linksted, Ingrid Kane, Will Whiteley, Robin Sellar, Philip White, Peter Keston, Andrew Farrell, Zoe Morris, Hector Miranda. **CTSU, Oxford**, Lisa Blackwell. **National Co-ordinating centres**: ***Italy*** up to September 2008: Maria Grazia Celani; Enrico Righetti. After September 2008: Silvia Cenciarelli; Tatiana Mazzoli. Central Follow up for Italy: Teresa Anna Cantisani. ***Poland*** Jan Bembenek. ***Sweden*** Eva Isaksson. ***Norway*** Eivind Berge, Karsten Bruins Slot.

List of members of IST-3 collaborative group and participating hospitals in each country.
Figures in parentheses are the number of patients recruited in the country or by the centre. ***UK*** *(1447)* Royal Hallamshire Hospital (118): G Venables, C Blank, H Bowler, C Doyle, K Endean, K Harkness, E Parker, M Randall. University Hospital of North Staffordshire (97): C Roffe, N Ahmad, A Arora, S Brammer, J Chembala, B Davies, S Ellis, E Epstein, K Finney, C Jackson, C Jadun, R Kinston, H Maguire, I Memon, I Natarajan, M Poulson, R Sanyal, S Sills, A Vreeburg, E Ward. Western General Hospital (95): P Sandercock, R Al-Shahi Salman, R Davenport, M Dennis, P Hand, S Hart, I Kane, S Keir, M MacLeod, L McKinlay, H Milligan, E Sandeman, J Stone, C Sudlow, P Taylor, J Wardlaw, C Warlow, W Whiteley, A Williams. The National Hospital for Neurology & Neurosurgery (84): M Brown, B Athwal, V Bassan, N Bhupathiraju, J Bowler, C Davie, D Doig, R Erande, S Gilbert, L Ginsberg, R Greenwood, S Gregoire, N Harding, N Losseff, R Luder, N Passeron, R Perry, P Rayson, R Simister, S Stone, D Werring. Arrowe Park Hospital (83): J Barrett, H Aitken, S Cherian, R Davis, S Downham, L Godd, V Gott, D Jose, V Little, D Lowe, L Luxford, M McGrory, P Owings, N Price, J Richards, G Sangster, J Sherlock, S Vargese, I Wakefield, P Weir. Southend University Hospital (77): P Guyler, T Attygale, S Chandler, L Coward, S Feasey, C Khuoge, T Loganathan, S Martin, A O'Brien, D Sinha, V Thompson, S Tysoe, R Walsh. Norfolk and Norwich University Hospital (67): K Metcalf, J Cochius, R Fulcher, N Gange, C Green, J Jagger, M Lee, P Myint, J Potter, G Ravenhill, S Shields, N Shinh, T Staunton, E Thomas, W Woodward, P Worth, N Wyatt. Nottingham City Hospital (63): W Sunman, P Bath, P Berman, J Clarke, C Gaynor, F Hammonds, R Harwood, K Mitchell, S Munshi, S Pacey, A Shetty, N Sprigg, H Stear, G Subramanian, A Wills. Guy's & St.Thomas Hospital (60): A Rudd, H Audebert, A Bhalla, J Birns, R Chowdhury, G Cluckie, I Davies, C Gibbs, P Holmes, N Mitchell, F Schiavone, E White, M Yeung. Darlington

and Bishop Auckland Hospitals (56): A Mehrzad, V Baliga, E Brown, L Burnside, B Esisi, J Kent, P Orr, D Stead, E Wayman. University Hospital Aintree (46): R Durairaj, C Cullen, R Kumar, H Martin, D McDowell, A Sharma, V Sutton, R White. University Hospital of Wales (46): T Hughes, K Ali, J Anderson, K Baker, K Bethune, K Bethune, M Booth, M Cossburn, S Halpin, M Hourihan, E Marsh, K Peall, R Powell, H Shetty, M Wardle, M Williams. Derby Royal Hospital (37): K Muhiddin, J Beavan, M Clarke, R Donneley, S Elliott, P Fox, P Gorman, M Harper, M Mangoyana, I Memon, L Mills, L Wright. Addenbrookes Hospital (34): L Warburton, J Baron, P Barry, D Day, T Harold, P Martin, J Mitchell, E O'Brien, J Rycarte, M Turnham. St George's Healthcare NHS Trust (34): G Cloud, L Choy, B Clarke, C Griffin, O Halse, I Jones, F Kennedy, U Khan, R Lewis, A Loosemore, C Lovelock, H Markus, B Moynihan, J O'Reilly, O Paul, A Pereira, M Punter, P Rich, D Rolfe, F Schiavone. Royal Devon & Exeter Hospital (Wonford) (30): M James, J Bell, A Bowring, L Boxall, J Cageao, H Eastwood, S Elyas, F Hall, S Harries, A Hemsley, S Jackson, S Keenan, P Mudd, A Sekhar, D Strain, J Sword, N Wedge. Aberdeen Royal Infirmary (26): M MacLeod, M Bruce, A Joyson, M Kemp, K McMullan, J Reid, O Robb, J Webster, S Wilkinson. Hammersmith Hospitals & Imperial College (24): P Sharma, P Bentley, H Jenkins, A Kar, T Sachs. Northwick Park Hospital (20): D Cohen, R Bathula, J Devine, M Mpelembue. William Harvey Hospital (20): D Hargroves, I Balogun, L Cowie, A Maidment, D Rand, J Rowe, H Rudenko, D Smithard, L Wray. Scarborough Hospital (17): J Paterson, J Brown, J Hampton, S Jamieson, R Rose, A Volans. Countess of Chester Hospital NHS Foundation Trust (17): K Chatterjee, G Abbott, R Brookes, C Castle, C Kelly, S Leason, A Nallasivan, A Sen. Watford General Hospital (17): D Collas, M Cottle, N Damani, P Jacob, D Oza, D Werring. University Hospitals Coventry & Warwickshire NHS Trust (15): A Kenton, N Adab, L Aldridge, H Allroggen, Y Brown, R Cross, L Galvin, K Ghosh, A Grubneac, A Lindahl, H Mehta, M Pritchard, C Randall, P Ray, A Shehu, S Thelwell. Royal Bournemouth & Christchurch NHS Trust (12): D Jenkinson, J Bell, T Black, O David, J Kwan, A Orpen, C Ovington, D Tiwari, Z ud Din Babar. Leeds General Infirmary (12): A Hassan, A Bailey, J Bamford, C Bedford, R Bellfield, J Cooper, L Dunsmure, J Greig, M Keeling, L Mandizvidza, J Rankine, E Roberts, P Wanklyn, T Webb, S Williamson. York Health Services NHS Trust (12): J Coyle, S Crane, C Croser, P Duffey, R Evans, E Iveson, M Keeling, G Kitching, M Porte, C Rhymes. Queen Elizabeth Hospital (Gateshead) (12): D Barer, M Armstrong, M Bokhari, T Cassidy, B McClelland. Queen Elizabeth The Queen Mother Hospital (10): G Gunathilagan, P DOLKE, S Jain, S Jones, A Maidment, L Rosser, G Thomas, C White. Worcestershire Royal Hospital (10): P Sanmuganathan, C Scholtz, E Stratford. Blackpool Victoria Hospital (10): M O'Donnell, H Goddard, G Hoadley, J Howard, S Leach, J McIlmoyle, A Stewart, A Strain. Basildon & Thurrock University Hospitals NHS FT (9): F Huwez, P Croot, N Gadi, N Mguni, U Umasankar. Royal Infirmary of Edinburgh (8): G Mead, B Chapman, A Coull, S Hart, A Kinnear, B Morrow, F Morrow. St Mary's Hospital (8): D Ames, J Ball, S Bannerjee, J Chataway. Yeovil District Hospital (8): K Rashed, C Buckley, D Donaldson, D Hayward, C Lawson. Luton and Dunstable Hospital (8): L Sekaran, K Bharaj, F Justin, G Jutlla, D Phiri, S Sethuraman, M Tate. Solihull Hospital, Heart of England NHS Trust (8): D Sandler, P Carr, G Jones, J Lyons, K Warren. King's College Hospital (7): L Kalra, A Davis, J Jarosz, D Manawadu, L Sztriha. Doncaster Royal Infirmary (7): D Chadha, A Holford, P Willcoxson. Royal United Hospital Bath (7): L Shaw, D Button, A Cunningham, L Dow, J Dutson, T Hall, C Hardy, N Jakeman, P Kaye, B Madigan, K O'Brien, D Pressdee, M Price, L Robinson, C Taylor, D Williamson. Birmingham Heartlands Hospital (6): D Sandler, P Carr, J Lyons, J McCormack, C Stretton. University Hospital North Durham (6): P Earnshaw, E Brown, S Bruce, C Church, S Desai, B Esisi, M Myint, N Watt. Wansbeck General Hospital (6): C Price, S Elliott, H Graham, R Lakey, K Mitchelson. Bristol Royal Infirmary (6): P Murphy, L Ball, S Caine, J Dovey, J Hughes, A Steele. Stepping Hill Hospital (6): K Dizayee, A Brown, T Chattopadhyay, J Cheetham, H Cochrane, A Datta, M Datta-chaudhuri, C Fox, D Kilroy, S Krishnamoorthy, F Levy, S Metha, P Ngoma, B Venkatesh. Princess Royal Hospital Brighton & Sussex University Hospitals Trust (5): K Ali, R Gautam, N Henderson, M Jones, S Murphy, G Spurling. Belfast City Hospital (5): I Wiggam, C Boyd, K Fullerton, P Gray, M Kinnaird, S MacNair, C Morgan, M Reid, S Tauro. Royal Liverpool University Hospital (5): S Loharuka, D Balmforth, P Cox, G Fletcher, A Ledger, A Manoj, M Wilkinson. City Hospital, Sandwell & West Birmingham Hospital (5): D Nicholl, S Clegg, S Hurdowar, S Kausar, K Law, A Singal, S Sturman. Royal London Hospital (4): P Gompertz, J Evanson, A Farrell, A Petrou, K Saastamoinen, T Sachs, A Salek-Haddadi, R Yadava. Sunderland Royal Hospital (4): J O'Connell, H Brew, S Butler, S Crawford, C Gray, D Gulliver, N Majmudar, R O'Brien. Morriston Hospital (4): M Wani, L Dacey, L Davies, R Evans, D Harris, T Jones, S Storton. Royal Preston Hospital (4): S Punekar, A Ashton, S Duberley, H Emsley, C Gilmour, B Gregary, L Hough, S Philip, S Wuppalapati. The Royal Wolverhampton Hospitals NHS Trust (4): K Fotherby, P Bourke, D D'Costa, K Kauldhar, D Leung, R Lodwick, S McBride, D Morgan, M Qaiyum, G Sahota, M Srinvasan. Royal West Sussex NHS Trust, St Richard's Hospital (4): I Kane, N Chuter, L Garrad, M Hookway, S Ivatts, G Kennedy. Queen's Hospital Romford (4): K Darawil, L Al Dhahirl, S Andole, M Baig, P Dugh, K Dunne, H Kariuki, M Khan, S Rathnayaka. Ulster Hospital (3): M Power, K Dynan, J Finnerty, A Heaney, C Leonard, K McKnight, J Turkington, B Wroath. Great Western Hospital (3): B Dewan, S Cotton, M Gardiner, T Saunders, B Vincent. The Queen Elizabeth Hospital Birmingham (3): D Sims, P Guest, E Jones, J McCormack, D Nicholl, J Savanhu, R Tongue,

M Willmot. Leicester General Hospital (3): D Eveson, S Dawson, M Dickens, M Fotherby, R Hunt, S Khan, T Kumar, R Marsh, A Mistri, T Robinson, J Thompson. Darent Valley Hospital, Dartford & Gravesham NHS Trust (3): P Aghoram, T Daniel, M Gatehouse, S Hussein, A Jackson, T Shanganya, E Strachan, G Tan. Nevill Hall Hospital, Aneurin Bevan Local Health (3): B Richard, S Elaine, S Hanson, S Mosely, H Reed, M Williams. Colchester Hospital University Foundation Trust (3): R Saksena, S Cook, D Demuran, M Keating, R Needle, V Paramsothy, A Sebastian, R Sivakumar, A Wright. Salford Royal Hospital Foundation NHS Trust (2): R Grue, E Barberan, C Dickson, C Douglas, J Jellicoe, T Marsden , J Priestley, E Quick, C Sherrington, A Singh, C Smith, J Stevens, P Tyrell, J Wainwright. Leicester Royal Infirmary (2): M Ardron, J Birchall. Queen Elizabeth Hospital (Kings Lynn) (2): R Shekhar, C Barsted, S Coleman, S Fletcher, J Graham. John Radcliffe Hospital (2): A Buchan, J Hinkle, J Kennedy, A Manoj, M Westwood. Derriford Hospital (2): A Mohd Nor, S Allder, B Hyams, A Pace. West Cumberland Hospital (1): E Orugun, C Brewer, L Huntley, R Jolly, C Summers. Sandwell General Hospital (1): K Sharobeem, J Khaira, J Leahy, E Linehan, G Moore, J Rizkalla, J Wilkinson. Torbay Hospital (1): D Kelly, C Hilaire. Warrington & Halton Hospitals NHS Foundation Trust (1): O Otaiku, L Connell, G Delaney-Sagar, G James, L Lomax, D Matthew, J Simpson, H Whittle. Medway Maritime Hospital (1): S Sanmuganathan, S Burrows, A Mahmood. Southampton General Hospital (1): G Durward, S Barker, J Cantle, P Crawford, S Evans, V Pressly, N Weir. Victoria Hospital (1): V Cvoro, K McCormick. ***Poland*** *(347)* 2nd Department of Neurology, Institute of Psychiatry & Neurology (190): A Czlonkowska, J Bembenek, M Bilik, G Chabik, W Czepiel, J Dzierka, M Gluszkiewicz, K Grabska, B Janus-Laszuk, J Jedrzejewska, A Kobayashi, T Litwin, A Oskedra, A Piorkowska, M Skowronska, A Sliwinska, U Stepien. SPZZOZ w Sandomierzu (43): P Sobolewski, A Gajewska, M Grzesik, R Hatalska-Zerebiec, I Labudzka, B Loch, A Medrykowska, M Sledzinska, A Sobota, W Szczuchniak, G Wolak, I Zdyb. Medical University of Gdansk (35): W Nyka, D Gasecki, K Chwojnicki, A Gojska, B Karaszewski, G Kozera, M Kwarciany, M Nowak, M Swierkocka-Miastkowska , S Szczyrba, M Wisniewska, E Wnorowska. 1st Department of Neurologyt, Institute of Psychiatry & Neurology (25): P Richter, A Bochynska, M Chahwan, A Graban, R Rola. Military Medical Institute (24): A Stepien, B Brodacki, M Grotowska, J Kotowicz, J Staszewski, J Swistak, S Zaloga. Szpital Powiatowy (14): J Stoiñski, K Czajkowaka-Fornal, P Czubak, A Kaczor, J Kraska, E Nowakowska-Sledz, J Ozdoba-Rot, E Zawadzka. Szpital Specjalistyczny w Konskich (8): M Fudala, D Adamczyk, W Brola, I Guldzinska, K Kaluzny, M Kucharska-Lipowska, M Mosiolek, M Polewczyk, M Ziomek. Central University Hospital (7): G Opala, M Arkuszewski, M Kudlacik, P Malgorzata, M Swiat. SPSK im. Prof. W. Orlowskiego CMKP (1): U Fiszer, M Lenska-Mieciek. ***Italy*** *(326)* Ospedale Di Citta' di Castello (62): S Cenciarelli, A Barilaro, R Condurso, F Coppola, S Dioguardi, E Gallinella, A Mattioni, C Menichetti, S Ricci. Nuovo Ospedale Civile "S.Agostino-Estense" (40): F Casoni, M Bacchelli, M Cavazzuti, M Malagoli, A Zini. Ospedale Beato Giacomo Villa - Citta' della Pieve (36): G Benemio, M Celani, R Allegrucci, V Bondo, S Cupella, L Guerra, S Guerrieri, C Ottaviani, E Righetti, C Rossi, N Sacchi, M Scucchi, V Stefanini. Ospedale Di Branca (36): T Mazzoli, A Bigaroni, L Greco, R Paris, P Parise, S Ricci. A.O. Niguarda Ca'Granda (25): A Ciccone, L Basso, R Causarano, P Doneda, E Ferrante, A Gatti, A Guccione, A Gullo, F Imbesi, S Jann, R Marazzi, E Moro, C Motto, D Parodi , A Protti, M Riva, A Rosiello, I Santilli, R Sterzi, P Tiraboschi, G Venturelli. R.Guzzardi Hospital - Vittoria (RG) (19): F Iemolo, R Campagna, G Campagnolo, A Carnemolla, N D'Apico, G D'Asta, S Giannarita, A Giordano, E Sanzar. Ospedale Regionale di Aosta (17): E Bottacchi, S Cordera, G Corso, M Di Giovanni, G Giardini, C Lia, T Meloni, M Pesenti Campagnoni, P Tosi. Ospedale Sacro Cuore - Negrar Verona (12): A Adami, G Rossato, T Zuppini. Ospedale Maggiore - Bologna (12): G Procaccianti, T Sacquegna. Universita degli Studi di Genova (11): C Gandolfo, M Balestrino, C Bruno, L Castellan, M Del Sette, A Ferrari, C Finocchi, N Reale, D Rizzi. Ospedale Civlle S.Andrea (9): M Del Sette, L Benedetti, C Capellini, E Carabelli, E Cibei, M Godani, G Guariglia, E Landini, A Mannironi, B Nucciarone, S Parodi, S Tonelli, E Traverso, D Zito. Ospedale S. Giovanni Battista - Foligno (8): P Brustenghi, F Corea, O Flamini, S Lolli, G Pelliccia, R Ricci, S Stefanucci, M Zampolini. Ospedale a Vibo Valentia (8): D Consoli, F Galati, P Postorino. Azienda Ospedaliero-Universitaria "Ospedali Riuniti" di Foggia (8): G Rinaldi, E Carapelle, G Grilli, M Guido, L Specchio. Ospedale Valduce di Como (8): N Checcarelli, G Borin, L Chiveri, R Clerici, E Corengia, L Gandola, P Garavaglia, M Guidotti, A Martegani, M Mauri, F Muscia, F Raudino. Ospedale di Cattinara - Trieste (4): F Chiodo Grandi, A Bratina, N Carraro, M Gaio, A Granato, N Koscica, M Naccarato, V Sarra, P Schincariol, C Vilotti, Z Zugna. Clinica Dr Pederzoli Spa (4): D Idone, C Bonato, E De Angelis, A Forgione, M Gambera, F Recchia, S Tamburin, P Tinazzi Martini, G Zanette. Ospedale Civile San Matteo Degli Infermi - Spoleto (2): S Grasselli. Ospedale Silvestrini - Perugia (2): G Agnelli, A Andrea, A Billecia, V Caso, V Casso, R Fabiola, P Fanelli, M Paciaroni, B Sergio, M Vemti. Mater Salutis Hospital, Legnago VR (2): M Silvestri, L Altarini, A Bonfante, M Bonornetti, B Costa, N D'Attoma, N Deluca, F Frattini, R Niego, D Rafaele, V Ravenna, M Turazzini. Ospedale Guglielmo da Saliceto - Piacenza (1): S Cammarata. ***Sweden*** *(297)* Uppsala University Hospital (100): E Lundström, L Jonsson , U Söderström, A Terént. Danderyd Hospital (46): V Murray, A Alvelius, M Arbin von, I Dalenbring, Å Doverhall, Å Franzén-Dahlin, N Greilert, M Hallberg, A Heijne von, E Isaksson, H Kumpulainen, A Laska, A Lundström, C Martin, J Muhrbeck, E Näslund, N Ringart, E Rooth, R Undén, P Waldenström. Hassleholm Hospital (29): M

Esbjornsson, M Petranek. Karnsjukhuset (25): B Cederin, E Bertholds, A Elgåsen, T Johansson, B Witteborn. Koping Hospital (20): M Kwiatkowska, E Gustafsson, T Noren, J Saaf. Mora Hospital (17): J Teichert, M Bertilsson, S Nilsson, S Oestberg. LidkopingHospital (11): L Welin, K Fredricson, L Pehn. Falu Hospital (11): J Hambraeus, I Lonn. Capio S: tGoran Hospital (9): B Hojeberg, A Adolfsson, M Anzen. Vastervik Hospital (5): T Wallen, R Schloenzig, P Söderström, A Wennerberg. University Hospital MAS (5): F Buchwald, K Abul-Kasim, A Berkeskold, J Petersson, E Poromaa. University Hospital of Northern Sweden (4): P Wester, R Backlund, A Sjöström. Helsingborgs lasarett (4): B Hedström , E Campbell, K Johnsson, B Karlsson, N Lekokotla, C Lundahl, A Risedal, P Sandgren, A Svensson. Visby Hospital (4): S Bysell, E Smedberg, A Vestberg Bysell. Sundsvall Hospital (3): V Sjögren, B Högvall. University Hospital Lund (2): G Andsberg, T Cronberg, A Lindgren. Vasteras Hospital (1): H Wannberg, F Ax, L Nyren. Karlstad Central Hospital (1): J Sanner, H Andersson, F Andler, S Holmgård, R Johansson, I Magnussan, K Nilsson, J Rådberg. ***Norway*** *(204)* Trondheim University Hospital (69): B Indredavik, H Ellekjær, A Østvik, G Rohweder, D Steckhan, J Storvold. Oslo University Hospital Sykehus (66): E Berge, Y Rønning, R Aakvik, K Bruins Slot, G Knutsen, M Moxness, R Pettersen, T Wyller. University Hospital of North Norway (23): C Wahl, O Iversen, S Johnsen, B Norderhus, L Steffensen, E Stensland. Kongsvinger Hospital (13): T Asak, J Aaseth, T Rotnes, J Sparby, S Wetterhus. Levanger Hospital (9): H Hallan, A Aardal, T Graven, H Hansbakk Skjetne, B Klykken, K Lindqvist, A Tommy. University Hospital of North Norway (8): T Engstad, M Antonsen, R Bajic, W Fønnebø, S Hykkerud, I Lyngmo, A Nyrnes, S Rogne, S Sparr. Harstad Hospital (7): O Kildahl-Andersen, K Pedersen, H Ulrichsen. Ålesund Hospital (4): O Skogen, I Alnes, R Hukari, Y Seljeseth, P Vadset. Asker and Bærum Hospital (2): G Knutsen, B Fure, H Ihle-Hansen, N Johnsen, L Kornberg. Namsos Hospital (2): S Schuler, M Heibert. Volda Hospital (1): M Lillebø, O Aasen, I Eskeland, T Hamre, S Hareide, H Helset, K Kolnes, B Lødemel, H Ose Velle, S Reite, E Velle. ***Australia*** *(179)* Nambour General Hospital (51): R Grimley, E Ahern, C Cocks, M Courtney, R Devin, J Endacott , C Fawcett , V Harrington, C Johnston, M Koltermann, S Murray, K Ng, G Styles, A Tampiyappa. John Hunter Hospital (29): C Levi, K Chung, L Dark, M Evans, Y Gawarikar, E Kerr, A Loiselle, F Miteff, A Moore, W O'Brien, M Parsons, D Quain, A Royan, M Russell, N Spratt. Gosford Hospital (24): J Sturm, D Crimmins, D Griffiths, P Kavelieros, J Kinsella, A Malhotra, B O'Brien, A Schutz, M Webb, S Whyte, V Zenteno. Westmead Hospital (16): R Lindley, A Bleasel, N Cordato, A Duggins, V Fung, L Gomes, N Ingham, J Ip, P Landau, J Morris, S Vucic. Royal Perth Hospital (15): G Hankey, A Claxton, N Lillywhite. The Canberra Hospital (12): C Lueck, C Andrews, G Danta, C Das, I Harvey, A Hughes, C McColl, A Oon, R Tuck. Royal Brisbane and Women's Hospital (10): S Read, M Badve , M Broad, G Cadigan, H Cavanagh, J Chalk, D Copsinis, K Etherington, R Henderson, R Hull, J O'Sullivan, J Pandian, L Ross-Lee, M Roxas, N Sheikh, G Skinner, A Wong. Austin Health - Repatriation Campus (8): H Dewey, A Brodtmann, G Donnan, A Hughes, M Karonen, H Ma, T Mulcahy, S Petrolo, L Walker, D Young, J Zavala. Nepean Hospital (7): M Thieben, C Harris, M Krause, S Lane, H Park, M Shaffi, J Wood. Box Hill Hospital (7): C Bladin, A Buckland, K Coughlan, B Coulton, A Gilligan, P Lee, S Mullen, Z Ross, P Sien Loh, C Szoeke. ***Portugal*** *(82)* UAVC. Centro Hospitalar de Trás-os-Montes e Alto Douro (42): M Silva, F Afonso, J Gabriel, P Guimarães, A Velon. Hospital Pero da Covilhã (19): M Castelo- Branco, F Alvarez, V Branco, C Coxo, P Goulao, D Leal, S Morgado, R Oliveira, F Paiva, A Rodrigues, M Simoes. Hospital de Santo António (12): G Lopes, T Almeida, M Cardoso, J Chaves, C Correia, M Correia, J Damásio, R Felgueiras, J Pereira, A Tuna. Hospital S.Marcos (9): C Ferreira, E Lourenco, A Machado, R Mare, J Rocha. ***Belgium*** *(73)* Cliniques Universitaires St Luc (73): A Peeters. ***Austria*** *(46)* Landesklinikum Donauregion Tulln (34): K Matz, M Brainin, G Funk, V Reiner-Deitemyer. Krankenhaus Der Barmherzigen Bruder Wien (9): J Ferrari, A Flamm-Horak, G Gruber, R Rattinger. Krankenhaus Göttlicher Heiland (3): W Muellbacher, D Doppelbauer, R Kalchmayr, W Schima, T Wieser, M Zart. ***Switzerland*** *(23)* Universitatsspital Basel (22): P Lyrer, L Bonati, S Engelter, F Fluri, S Muller, E Radue, A Tiemessen, L Walz, F Weisskopf, S Wetzel. Universitätsspital Zürich (1): A Luft, D Fetz, B Hertler, A Pangalu. ***Canada*** *(8)* QEII Health Sciences Centre (8): G Gubitz, P Boulton, J Jarrett, J Moeller, S Phillips. ***Mexico*** *(3)* Instituto Nacional de Neurologia y Neurojrugia MVS (3): A Arauz, L Bermudez, J Calleja, R Garcia.
